# Metabolomics for the Identification of Biomarkers in Kidney Diseases

**DOI:** 10.7150/ntno.108320

**Published:** 2025-03-24

**Authors:** Swarnima Pandey

**Affiliations:** University of Maryland, School of Pharmacy, Department of Pharmaceutical Sciences, Baltimore, MD, USA.

**Keywords:** metabolomics, diabetes, kidney, cardiovascular, critical illness

## Abstract

With the apparent rise in lifestyle-related changes, there has been a significant decline in renal health. Metabolomics plays a crucial role in the prognosis, diagnosis, and treatment of various renal conditions, including chronic kidney disease, acute kidney injury, diabetic kidney disease, kidney cancer, and post-transplant complications. Metabolomics has identified novel biomarkers, providing insights into altered pathways and potential therapeutic targets for kidney diseases. Kidney diseases and metabolomics keywords were searched in correspondence with the assigned keywords, including chronic kidney diseases, acute kidney injury, kidney carcinoma, kidney transplant, and diabetic kidney diseases on literature search engines. The applicable studies from this search were extracted and included in the study. This review is focused on the biomarkers identified in different kidney diseases such as chronic kidney diseases, acute kidney injury, diabetic kidney disease, kidney carcinoma and kidney transplant.

## Introduction

**Figure 1:**
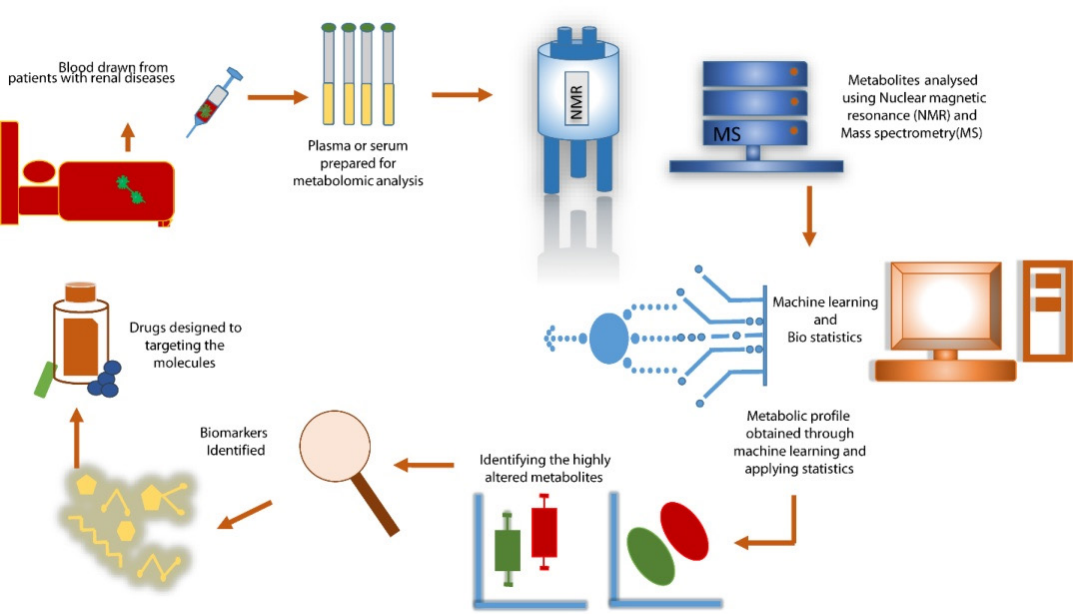
Metabolomics Workflow in Kidney Diseases. The metabolomics approach measures metabolic responses in patients with kidney diseases using biological fluids like serum. Advanced analytical techniques, such as nuclear magnetic resonance (NMR) and mass spectrometry (MS), are utilized to detect and quantify metabolites. Statistical analysis of the data identifies target metabolites associated with kidney conditions, aiding in the understanding of pathophysiological mechanisms, diagnosis, prognosis, and therapeutic interventions.

**Figure 2:**
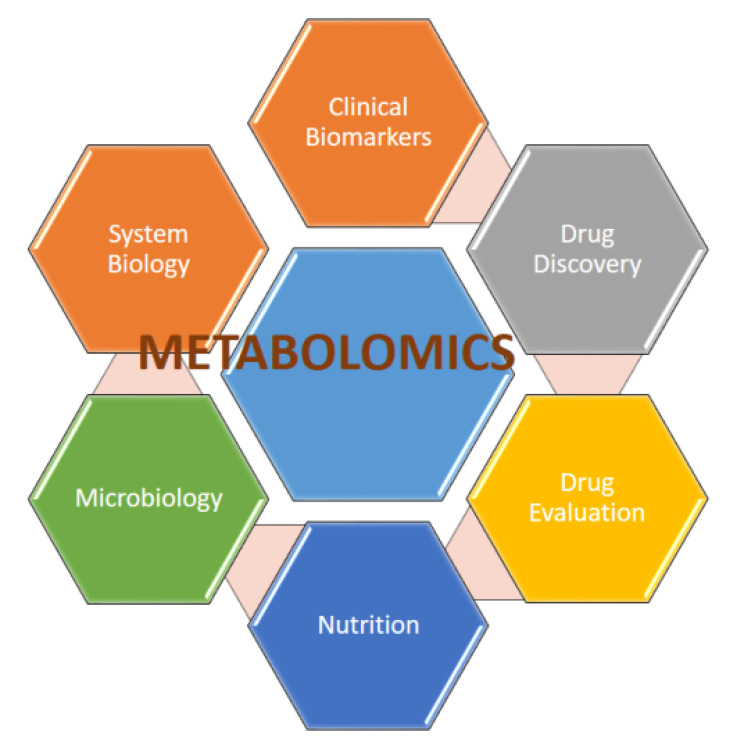
Clinical Applications of Metabolomics in Kidney Diseases. Metabolomics plays a pivotal role in advancing kidney disease research and management. Its applications include identifying biomarkers for early detection, differentiating between disease types (e.g., CKD, AKI, DKD), predicting disease progression, and monitoring responses to therapy. Additionally, metabolomics contributes to uncovering molecular pathways involved in kidney disease pathophysiology, enabling precision medicine and improved patient care.

Omics sciences have recently evolved into the most potent approach for identifying biomarkers for various diseases, from lifestyle disorders like diabetes and obesity to nephropathy to critical illnesses such as sepsis 1-13. The molecular approach following these metabolites would give a broader picture of the role of these metabolites in various pathways in multiple diseases 14, 15. Metabolomics provides insight into the pathophysiological mechanism of the broad spectrum of renal diseases (acute kidney injury, chronic kidney disease, diabetic nephropathy, polycystic kidney disease, renal carcinoma, and kidney transplant) 15,16 (Figure 1). Metabolomics could aid in management of the complex kidney conditions (Figure 2). An estimated 700 million individuals worldwide are living with CKD^17^.Acute kidney injury (AKI) is a significant global health concern, particularly prevalent among hospitalized patients. In pediatric trauma patients in Malawi, AKI occurs in up to 10% of admissions, increasing the risk of death sevenfold compared to those without AKI 18.DKD is the leading cause of end-stage renal disease (ESRD) in the U.S., accounting for about 50% of new cases^19^.Kidney cancer accounts for approximately 2% of global cancer diagnoses and deaths. In 2020, there were about 400,000 new cases and 180,000 deaths attributed to renal cell carcinoma worldwide^20^.In 2019, the U.S. performed a record 24,273 kidney transplants, with approximately 72% from deceased donors. As of December 2018, there were 229,887 patients in the U.S. with a functioning kidney transplant, representing a 40% growth since 2008^21^.

Metabolomics is not yet fully integrated into routine clinical nephrology. A review can advocate for its broader adoption by summarizing the benefits and potential applications in diagnosis, prognosis, and personalized treatments. It can identify gaps in research, such as limitations in biomarker discovery, challenges in clinical translation, or areas where metabolomics has been underutilized, guiding future studies. This review focuses on the application of metabolomics in kidney diseases, which encompass conditions such as acute kidney injury, chronic kidney disease, diabetic nephropathy, polycystic kidney disease, renal carcinoma, and complications arising from kidney transplantation. By elucidating the metabolic alterations associated with these diseases, metabolomics offers valuable insights into pathophysiological mechanisms, aiding in the diagnosis, prognosis, and management of complex renal conditions.

The purpose of this review is threefold: (1) to summarize recent advances in the identification of biomarkers for various kidney diseases using metabolomics, (2) to highlight gaps and challenges in the current research landscape, and (3) to propose future directions for leveraging metabolomics to improve clinical outcomes. By synthesizing findings from a wide range of studies, we aim to provide a comprehensive understanding of how metabolomics can address unmet needs in renal disease research and patient care. Furthermore, this review emphasizes the translational potential of metabolomic biomarkers in paving the way for personalized medicine in nephrology.

## Chronic Kidney Disease

There are several potential biomarkers of diagnostic and prognostic potential reported for chronic kidney disease (CKD) such as alanine, valine, glutamine, glycine, arginine, proline, glucose, lactate, succinate, fumarate, betaine, myoinositol, taurine, TMAO, glycerophosphocholine, indoxyl sulfate, adenosine monophosphate (AMP), and guanosine monophosphate (GMP) 22-24.

Untargeted metabolomics performed on large-scale studies involving 1-5 stages of CKD and healthy control enlisted taurine, tiglylcarnitine, canavaninosuccinate, acetylcarnitine, and 5-MTP, as potential biomarkers 25. Among these, 5-MTP was reported to be closely related to the development and progression of kidney disease. Moreover, 5-MTP as a therapeutical target enhanced the keap1/Nrf2 signaling pathway and suppressed the IκB/NF-κB signaling pathway 25. Major differences in metabolite profiles with increasing stage of CKD were observed, including altered arginine metabolism, elevated coagulation/inflammation, impaired carboxylate anion transport, and decreased adrenal steroid hormone production.^26^.

One of the after-effects of CKD is reduced sensitivity to insulin. A study involving 95 non-diabetic patient plasma samples who had undergone hyperinsulinaemic-euglycaemic clamp 27 illustrated tryptophan metabolism, TCA cycle, and ubiquinone biosynthesis as primary aberrations between CKD and control.

Zhang *et al.* identified biomarkers of CKD such as including lysophosphatidic acid (LPA) (18:2), cytosine, stearic acid, ricinoleic acid, arginine acid, LPA (16:0) and 3-methylhistidine 28. Apart from serum creatinine, nine other metabolites can predict CKD better 29. Of these, choline and citrulline were identified as markers of kidney metabolism^30^. D-asparagine and D-serine were reported to be highly related to CKD progression.

Another aspect of imbalance in renal osmotic pressure regulation would increase renal cell damage, aggravating CKD. Hence, increased urinary inositol and betaine levels are prognostic markers for CKD progression^31^. Another study performed in the African American population identified 5-oxo proline and 1,5-anhydroglucitol to be correlated with a lower risk of CKD 32,33.

Goek *et al.* identified spermidine and some metabolite ratios such as phosphatidylcholine diacyl C42:5-to-phosphatidylcholine acyl-alkyl C36:0 ratio and kynurenine-to-tryptophan ratio to be associated with glomerular filtration rate 34.

The TCA cycle intermediates play an important role in CKD. Hallan *et al.* identified urinary TCA intermediates such as succinate and isocitrate, to be decreased by 40-68%, while 2-oxoglutarate and citrate excretion was significantly increased in patients with CKD 35.

Another study including three cohorts: a Chronic Renal Insufficiency Cohort (CRIC) study, an African-American Study of Kidney Disease and Hypertension (AASK), and an Atherosclerosis Risk in Communities (ARIC) analysis was performed to identify pseudouridine, methylimidazoleacetate, and homocitrulline -- associated with CKD progression in CRIC, AASK, and ARIC. Three kynurenine derivatives, namely, 2-aminobenzoic acid, xanthurenic acid, and hydroxypicolinic acid, were upregulated in ESRD compared to CKD with the progression of CKD to ESRD 36.

Zhu *et al.* performed metabolomics evaluation of patients with stage 5 chronic kidney disease before dialysis, maintenance hemodialysis, and peritoneal dialysis to identify 20 discriminating metabolites screened from the group comparisons, including 2-keto-D-gluconic acid, kynurenic acid, s-adenosylhomocysteine, L-glutamine, adenosine, and nicotinamide^37^.

Works of Peng *et al.* identified metabolites highly correlated with kidney function, ranked from the highest down 38. These features were creatinine, N-acetylglucosamine/N-acetylgalactosamine, corticosterone, cis-3,4-methyl gamma-glutamylglutamine, 7-methylguanine, alanine, phenylalanylhydroxyproline, hydroxyasparagine, gamma-glutamyl-isoleucine, undecylenoyl carnitine (C11:1), trimethylamine N-oxide, N-acetylserine, sphingomyelin (d18:0/18:0, d19:0/17:0), pseudouridine, epiandrosterone sulfate, 5-methylthioribose, glutamine_degradant, 1-(1-enyl-palmitoyl)-2-arachidonoyl-GPC (P-16:0/20:4), 5,6-dihydrouridine, N6-carbamoylthreonyladenosine, N,N-dimethyl-pro-pro, 1-methylguanidine, retino (vitamin A), 3-(3-amino-3-carboxyproxypropyl)uridine, erythronate, 1-(1-enyl-palmitoyl)-GPC (P-16:0), gulonate, arabitol/xylitol, and C-glycosyltrptophan.

L-serine, Galacturonic acid, L-glutamine and lower concentrations of Pseudo uridine penta-tms, Butanoic acid, 2,4-bis[(trimethylsilyl)oxy]-, trimethylsilyl ester, 2-O-Glycerol-.alpha.-d-galactopyranoside, hexa-TMS, Myo-inositol, p-cresol, Lactose were significantly altered in patients who did not survive CKD^39^. Yong *et al.* identified that trimethylamine N-oxide delays the CKD 40.

Lipidomics helped in the identification of lipids altered in CKD and its progression. Glycerophospholipids, glycerolipids, and total fatty acids in serum were positively related to the increase in serum triglycerides and negatively associated with eGFR 41. Renal tubular epithelial cells were the main site of toxin-induced lipid accumulation, and lipidomics showed that turn-over effectively reduced the levels of phosphatidylcholines, triglycerides, and phosphatidylethanolamines in nephropathy, providing novel insights into the underlying mechanism of toxin-induced lipotoxicity in renal tubular epithelial cells 42. Metabolomics using Nuclear magnetic resonance reported Metabolomics using Nuclear magnetic resonance very-low-density lipoproteins, high-density lipoproteins, the lipid concentration and composition within these lipoproteins, triglycerides within all the lipoprotein subclasses, fatty acids, amino acids, and inflammation biomarkers were associated with CKD risk 43.

## Acute Kidney Injury

In a study with patients who received transcatheter aortic valve replacement (TAVR) 44, that included AKI patients as per the definition 45,46. The study had a total of 44 patients, 22 with CKD and nine developed AKI. The study reported that baseline levels of 5-adenosylhomocysteine predicted AKI after TAVR, despite adjustment for baseline glomerular filtration rate. Another study involving 2164 patients identified six metabolites that correlated with baseline estimated glomerular filtration rate (eGFR) 47. Lastly, 5--adenosylhomocysteine was identified to correlate with the dynamic changes in serum creatinine and the predictive potential for AKI. The AKI probability increased with the increase of 5- adenosylhomocysteine.

A single-center study by Zhang *et al.* on kidney transplant^48^ states that of the 42 transplants, 22 had developed AKI. Targeted metabolomic analysis was performed to identify tryptophan and arginine as highly altered. Tryptophan concentration was lower in AKI patients than in transplant patients without AKI. The prediction analysis identified Symmetric dimethylarginine (SDMA) and tryptophan together with an AUC of 0.9.

The LC-MS/MS-based non-targeted metabolomics method was used to differentially screen serum and urine metabolites of acute kidney injury (AKI) patients and healthy people by Chen *et al.* 49. Blood and urine were collected from 30 AKI subjects and 20 healthy controls. Urine profiling identified 2-S-glutathione glutathione acetate, 5-l-glutamyl-taurine, and l-phosphoarginine with a positive correlation for serum creatinine.

Vancomycin-associated AKI (VAKI) was studied by Lee *et al.*, including the study group along with patients who received vancomycin for infection control but who did not acquire AKI (non-VAKI), chronic kidney disease subjects, and 23 healthy persons 50. Serotonin (5-HT) and 5-hydroxy indole acetic acid (5-HIAA) were significantly higher and lower in the VAKI group than in the non-VAKI groups, respectively.

Another study established the 5-HIAA/5-HT ratio as a biomarker for VAKI 51. The study was performed in urine samples of 159 coronary artery bypass surgery patients to identify the metabolites predictive of developing AKI post-cardiac surgery subjects. AKI predictive metabolites were identified such as tyrosyl-gamma-glutamate, deoxycholic acid glycine conjugate, 5-acetylamino-6-amino-3- methyluracil, arginyl-arginine, and L-methionine.

Urine samples were collected from children: pre-AKI, established AKI and controls 52,53. Another study in pediatric ICU patients was performed to identify biomarkers for early diagnosis and grading 54. The urine samples were collected a maximum of three days post admission until days 5 or 14. The study revealed 20 metabolites as pre AKI and 13 metabolites with great potential for AKI prediction upto three days before AKI onset.

Gisewhite *et al.* utilized nuclear magnetic resonance to identify mortality/RRT (renal replacement therapy) and AKI stage. They identified metabolites that were correlated with mortality and AKI 1-Methylnicotinamide was common in all the outcomes and highest as the mortality outcomes. Lactate and 1-methyl nicotinamide levels were found in adverse AKI stages and RRT whereas glycine was reported to be associated with survival that did not require RRT 55.

Sun *et al.* demonstrated that at the early stage of RRT, serum metabolic biomarkers might differentiate patients with a high risk of mortality or permanent kidney injury from those who can recover 56. In the early days 1-arachidonoyl-lysoPC and 1-eicosatetraenoyl-lysoPC were significant. Jun *et al.* performed a meta-analysis to reveal that more intense RRT was independent of mortality risk 57.

## Diabetic Kidney Disease

Early biomarkers with the potential to identify the onset of diabetic kidney disease (DKD) would aid in preventing or delaying the DKD progression. Various metabolomics techniques have been used to investigate and identify the biomarkers of DKD, providing insight into the metabolic pathways obliterated, leading to the development of DKD. The following helps characterize the biomarkers of DKD.

Besides mass spectrometry, people use nuclear magnetic resonance (NMR) to perform metabolic studies. One of the NMR-based studies reports that lower levels of leucine and isoleucine are found in baseline eGFR patients with type 2 58 BCAA is identified as a prognosis marker in type 1 diabetes 59. Whereas Leucine and valine were identified as mortality markers in patients with diabetes 60. Metabolites associated with a low and high risk of rapid eGFR decline are glycine and n-acetlythreonine, respectively 61,62. 3-hydroxybutyrate is an important metabolite in DKD and ESKD 63,64.

Organic acids like uracil were altered in urine samples of patients with DKD 63,65. The urinary albumin-creatinine ratio increased in patients with T2D 62 and end-stage kidney disease (ESKD) 66,67. Alpha-hydroxybutyrate is positively associated with insulin resistance and diabetes but negatively associated with ESKD in patients with diabetes 66. Aliphatic amino acids such as glycine are found to have decreased levels in patients with DKD 68, along with glycolic acid associated with ESKD 63,64,68. Acetoacetate is reported to be negatively associated with baseline eGFR in patients with diabetes, and methyl acetoacetate was reduced in patients with DKD 63. The organic acids mentioned above are involved in energy metabolism, suggesting a dysregulation of mitochondrial function in DKD.

A higher level of SDMA has been found in people with DKD 69. The ratio of SDMA to ADMA is predictive of the decline of kidney function in diabetes 70,71. At the same time, ADMA has been associated with rapid kidney function decline in diabetes 71.

Aromatic amino acids like tryptophan and kynurenine are reported to be obliterated in DKD 61,62,66,67,70,72. Pawlak *et al.* said that the upregulation of tryptophan metabolism is correlated with impaired kidney function 73. High serum levels of tryptophan are inversely correlated with rapid eGFR decline in patients with DKD 61,70. Likewise, elevated levels of downstream metabolites of tryptophan were directly associated with DKD 62,66,67.

Lipoproteins like HDL (High-density lipoproteins) in relation to baseline eGFR were studied in several diabetic cohorts using NMR, illustrating that they are associated with a higher baseline of eGFR. On the contrary, triglycerides-rich lipoproteins were inversely correlated with albuminuria 58,74. A study involving the Global Lipids Genetics Consortium (n = 188,577) and the CKD Genetics Consortium (n = 133,814) reported that HDL cholesterol was associated lower risk of CKD 75.

Phospholipids such as Phosphatidylcholine (PC) and phosphatidylethanolamine (PE) have been reported with DKD. In a case-control study, they reported a lower level of PCs in patients with diabetes and CKD^76^. Another survey by Cooperative Health Research in the Region of Augsburg (KORA) stated that serum PCs is predictive of CKD in diabetic patients 77. Unsaturated PEs could differentiate between the progressors and non-progressors of diabetes kidney disease in patients with diabetes 78.

Higher ceramide metabolites have been demonstrated in patients with DKD 76,79. Similarly, elevated levels of sphingomyelin are found in patients with DKD 80, progression of DKD in patients with diabetes^81^, and chances of developing CKD in hyperglycemic patients^77^.

Afshinnia *et al.* reported that in patients with diabetes and baseline eGFR, FFAs are associated with a lower risk of eGFR reduction^78^. Β-oxidation of Fatty acids results in acylcarnitines, which are elevated in DKD^76^. Independent of other risk factors, C-16 acylcarnitines are among the strongest predictors of fast eGFR decline in patients with diabetes and CKD^70^. As with the amino acids, dysregulation of lipid metabolism indicates disturbed energy metabolism, thus mitochondrial dysfunction in the development of DKD.

Early biomarkers with the potential to identify the onset of diabetic kidney disease (DKD) would aid in preventing or delaying DKD progression. Metabolomics has proven instrumental in identifying these early-stage biomarkers, which provide crucial insights into disrupted metabolic pathways long before significant clinical manifestations appear. By utilizing techniques such as mass spectrometry and nuclear magnetic resonance (NMR), researchers have detected alterations in amino acids, organic acids, lipids, and other metabolites that signal the early onset of DKD. For example, lower levels of leucine and isoleucine, markers of impaired mitochondrial function, have been observed in baseline eGFR patients with type 2 diabetes, while metabolites such as glycine and N-acetylthreonine have been linked to rapid declines in renal function.

The ability of metabolomics to reveal these biomarkers facilitates timely intervention strategies, enabling healthcare providers to implement targeted therapies and lifestyle modifications to mitigate disease progression. Furthermore, the identification of dysregulated pathways--such as those related to energy metabolism, oxidative stress, and inflammation--provides opportunities to develop novel therapeutic targets. This proactive approach underscores the potential of metabolomics not only in diagnosing DKD but also in informing preventive measures and personalized treatment plans, ultimately improving patient outcomes.

## Kidney Carcinoma

Pyruvate is converted to lactate in RCC patients, contrary to healthy control 82. These metabolites were also elevated in tumor tissues 83-85 and urine samples 86. Priolo *et al.* identified fumarate, malate, and citrate as potential biomarkers in 87, 88,89.

Glutathione is elevated in cancer progression 90, including RCC 91. Hakimi *et al.* studied that patients with high glutathione levels were likely to progress into advanced stages. Apart from that α-hydroxybutyrate, it was significant in distinguishing stage II patients. Since the role of glutathione metabolism impairment is critical in the pathogenesis of RCC, this could be targeted for therapeutics 87.

Apart from glutathione, glutamine is also elevated in renal tissues 83. Fu *et al.* targeted the RCC with elevated glutamine as the therapeutic target to establish that Interleukin 23 (IL-23) enhanced cancer metastasis via elevated regulatory T cells 92. Patients with high IL-23 had a lower survival rate than patients with lower IL-23 92. Glutaminase inhibitor (CB-839) is being utilized for RCC treatment with good efficacy 93. Work by Gao *et al.* reported that amino acids like alanine and valine were decreased in RCC patients.

Alanine, choline, creatine, lactate, isoleucine, leucine, and valine had diagnostic potential for RCC, and the efficacy of these amino acids was validated in 22 additional independent subjects 82. Apart from these amino acids, metabolites of this amino acid metabolism, such as dentist acid, 4-hydroxybenzoic acid, and quinolinic acid, were altered in the urine samples of RCC patients 94.

Liu *et al.* identified a 1.67 and 2.07-fold increase in the levels of N-formylkynurenine in healthy control and benign tumors, respectively, identifying its potential as a biomarker for distinguishing RCC, benign renal tumors, and healthy control 95.

Vitamin E has been implicated in RCC prognosis 96. Sobotka *et al.* the prognostic potential of Vitamin E by studying 102 RCC patients and establishing an inverse relationship between survival and vitamin E 97. Similarly, Vitamin D has been shown to be associated with a lower risk for RCC in a study with 46,380 men and 72,051 women 98.

Sheila *et al.* identified various acylcarnitines to be closely associated with RCC grade. Acylcarnitines were reported to be upregulated in the urine of patients with high-grade cancer 99. Another study supported the role of acylcarnitine in RCC by demonstrating the elevated levels of acylcarnitine in the tissue and urine of RCC patients 100. RCC progression involves choline metabolism, as reported by Lin *et al.* 101, 102.

Ragone *et al.* identified that hippuric acid was decreased in the urine samples of RCC patients compared to the control 86. Yoshimura *et al.* established a novel diagnostic approach by utilizing electrospray ionization/ mass spectrometry to detect the boundary of cancerous regions by identifying the expression of triacylglycerols in 9 RCC patients 103.

## Kidney Transplantation

The development of minimally invasive method for kidney function in transplant is necessary. Hence, the metabolomics approach would be an appropriate one. Iwamoto *et al.* analyzed kidney transplant recipients' plasma, saliva, and urine 104. The study identified metabolic aberrations in T cell-mediated rejection (TCR), antibody-mediated rejection, and other kidney disorders (KD). The three biofluids showed different metabolic patterns between T cell-mediated rejection (TCR) and kidney disorders (KD), with 3-indoxyl sulfate showing a significant increase in TCR plasma and urine samples. The study demonstrated that 3-indoxyl sulfate had the potential to predict acute rejection. Urinary metabolomics for non-invasivenon-invasive detection of antibody-mediated rejection (AMR) identified proline, citrulline, phosphatidylcholine aa.C34.4, C10.2, and tetradecanoylcarnitine as diagnostic markers for AMR^105^.

Ivana *et al.* performed serum metabolomics in 19 individuals who received kidney transplants and collected samples before and after transplantation. The study identified hippurate, mannitol, and alanine as associated with the changes in renal function during the post-transplantation recovery period^106^. Colas *et al.* performed metabolomic studies in spontaneous tolerant kidney transplants that are long-term and functional grafts without immunosuppressive drug intake to identify tryptophan-derived metabolites such as kynurenic acid and tryptamine, characterizing the operational tolerance 107.

Dedinska *et al.* aimed to help with the non-invasive-invasive diagnosis of graft rejection due to T-cell activation by identifying metabolites reflecting the activation of T cells such as glutamine, lactate, tyrosine, and branched chain α keto acids (BCKAs)^108^. In all, a panel of nine differential metabolites was identified as novel potential metabolite biomarkers of T- cell-mediated rejection 109. Liu *et al.* performed a multicenter study to detect metabolites in perfusate collected at the beginning and end of deceased-donor kidney perfusion and evaluated their associations with graft failure. Alpha-ketoglutarate, 3-carboxy-4-methyl-5-propyl-2-furanpropanoate, 1-carboxyethyl phenylalanine, and three glycerolphosphatidylcholineswere found to be associated with increased graft rejection.^110^.

The kidney graft recovery process can be examined noninvasively by studying urine samples 111. Urinary metabolome of donation after circulatory death (DCD) transplant recipients. Revealed that branched-chain amino acids (BCAAs) over pyroglutamate and lactate over fumarate predicted prolonged functional delayed graft rejection (PDGF) 105. Chronic kidney allograft damage is characterized by arginine, histidine, proline, and SDMA 112. The lower level of serum dehydroepiandrosterone sulfate was found in the acute graft rejection group before transplantation^113^.

The assessment of kidney function within the first year following transplantation was performed via metabolomics using the non-targeted approach^114^. Nineteen metabolites were found to differ significantly in the 1^st^ week and seventeen in the 3^rd^ month; no significant differences were observed in the 6^th^ month^115^. Metabolomic profiling in individuals with a failing kidney allograft revealed choline, creatine, taurine, and threonine in individuals with lower GFR^116^.

Untargeted analysis of feces was performed using gas chromatography-mass spectrometry (GC-MS) to identify difference between kidney transplant and control group. The fecal metabolome was high in sterols and fatty acids in the stable transplant group 117.

Intestinal microbiome and metabolome analyses reveal metabolic disorders in the early stage of renal transplantation^118^,^119^.* Enterococcus* was found to be correlated with renal functions and metabolites reflecting renal damage 120.

## Key Metabolomic Biomarkers for Kidney Function

Following are the list of about some diagnostic biomarkers related to Kidney function impairments identified and established using metabolomics approach.

### A. Tryptophan Metabolism Pathway

1. Kynurenine

2. Kynurenic acid

3. 3-indoxyl sulfate

These metabolites are typically elevated in chronic kidney disease (CKD) and correlate with disease progression^34^,^36^,^73^.

### B. Gut Microbiome-Derived Metabolites

1. p-cresyl sulfate

2. Trimethylamine N-oxide (TMAO)

Indoxyl sulfate

These are uremic toxins that accumulate in kidney dysfunction 22,23,113.

### C. Acylcarnitines

1. Long-chain acylcarnitines

2. Short-chain acylcarnitines

Altered levels indicate disturbed fatty acid oxidation in kidney disease^70^,^99^.

### D. Amino Acid-Related

1. Symmetric dimethylarginine (SDMA)

2. Asymmetric dimethylarginine (ADMA)

These are particularly useful for early detection of kidney dysfunction^70^,^71^.

## Future Directions

The field of metabolomics is poised for significant advancements, driven by innovations in technology and computational tools. One promising avenue is the integration of machine learning (ML) with metabolomics data analysis. ML algorithms can process vast and complex datasets, uncovering subtle patterns and relationships that might otherwise remain undetected. This approach holds immense potential for refining biomarker discovery, enhancing diagnostic accuracy, and predicting disease progression with greater precision.

Another transformative development is single-cell metabolomics, which enables the analysis of metabolic profiles at the individual cell level. This technology could revolutionize kidney research by providing unprecedented insights into cellular heterogeneity and the microenvironment within renal tissues. For instance, single-cell metabolomics could help identify cell-specific metabolic alterations in kidney diseases, offering new perspectives on pathophysiology and potential therapeutic targets.

Additionally, advancements in real-time metabolomics and portable analytical devices may enable point-of-care testing, facilitating rapid and non-invasive diagnostics. The development of more sensitive and high-throughput platforms will also expand the scope of metabolomics studies, allowing for the comprehensive profiling of low-abundance metabolites and their dynamic changes during disease progression.

These technological innovations, combined with interdisciplinary collaboration and the growth of metabolomics databases, are set to propel the field forward. By leveraging these advancements, kidney research can move closer to achieving precision medicine, where interventions are tailored to the unique metabolic signatures of individual patients.

## Limitations

Despite its significant potential, metabolomics research faces several limitations that must be addressed to maximize its utility in kidney disease research. One major challenge is the small sample size of many studies, which limits the generalizability of findings and increases the risk of statistical bias. Expanding sample sizes and ensuring adequate statistical power are critical for deriving robust and reproducible results.

Population diversity is another limitation, as many metabolomics studies are conducted in homogenous populations that do not reflect global demographic and genetic variability. This lack of diversity hinders the applicability of findings to broader populations and underscores the need for more inclusive and representative study cohorts.

Reproducibility remains a persistent challenge in metabolomics research due to variations in experimental protocols, sample handling, and data analysis methods. Standardizing workflows, from sample preparation to data processing, is essential for improving reproducibility and enabling cross-study comparisons.

Additionally, metabolomics studies often struggle with the complexity of metabolite identification and quantification. Many metabolites remain unannotated, and their biological significance is poorly understood. Addressing this limitation will require advancements in computational tools, databases, and collaborative efforts to improve metabolite annotation and pathway mapping.

By addressing these limitations, future metabolomics studies can achieve greater reliability and impact, ultimately enhancing their contribution to understanding and managing kidney diseases.

## Conclusion

Metabolomics has provided great insight into many candidate metabolites appropriate as biomarkers for several kidney diseases and for deciphering the onset and progression of the diseases. Most metabolites and their pathways are linked to oxidative stress and inflammation. These interpretations are limited, many features remain unidentified. As the database grows, we can interrogate it further to understand kidney diseases. Clinicians can leverage metabolomics not only for diagnosis and treatment but also as a tool to enhance patient outcomes through early interventions, precision medicine, and a better understanding of disease mechanisms. By integrating these findings into clinical workflows, metabolomics has the potential to transform nephrology practice.

## Figures and Tables

**Table 1:** List of potential biomarkers of diagnostic and prognostic potential reported for chronic kidney disease (CKD).

Metabolite Class	Biomarker	Type	Metabolic Pathway
Amino Acids	Alanine	Diagnostic/Prognostic	Protein metabolism
	Valine	Diagnostic/Prognostic	Branched-chain amino acid metabolism
	Glutamine	Diagnostic/Prognostic	Nitrogen metabolism
	Glycine	Diagnostic/Prognostic	One-carbon metabolism
	Arginine	Diagnostic/Prognostic	Urea cycle
	Proline	Diagnostic/Prognostic	Protein metabolism
Energy Metabolites	Glucose	Diagnostic	Carbohydrate metabolism
	Lactate	Diagnostic	Anaerobic metabolism
TCA Cycle Intermediates	Succinate	Diagnostic	Energy metabolism
	Fumarate	Diagnostic	Energy metabolism
Osmolytes	Betaine	Diagnostic	Methionine metabolism
	Myoinositol	Diagnostic	Osmotic regulation
	Taurine	Diagnostic	Sulfur metabolism
Gut Microbiome-Derived	TMAO	Prognostic	Gut microbiome metabolism
Phospholipid-Related	Glycerophosphocholine	Diagnostic	Membrane metabolism
Uremic Toxins	Indoxyl sulfate	Prognostic	Protein metabolism
Nucleotides	AMP	Diagnostic	Purine metabolism
	GMP	Diagnostic	Purine metabolism
